# Chronic Obstructive Pulmonary Disease: Epidemiology, Biomarkers, and Paving the Way to Lung Cancer

**DOI:** 10.3390/jcm10132889

**Published:** 2021-06-29

**Authors:** Klára Szalontai, Nikolett Gémes, József Furák, Tünde Varga, Patrícia Neuperger, József Á. Balog, László G. Puskás, Gábor J. Szebeni

**Affiliations:** 1 Csongrád County Hospital of Chest Diseases, Alkotmány u. 36., H6772 Deszk, Hungary; szalontai@deszkikorhaz.hu; 2 Laboratory of Functional Genomics, Biological Research Centre, Temesvári krt. 62., H6726 Szeged, Hungary; gemes.nikolett@brc.hu (N.G.); vartun32@gmail.com (T.V.); neuperger.patricia@brc.hu (P.N.); balog.jozsef@brc.hu (J.Á.B.); laszlo@avidinbiotech.com (L.G.P.); 3 PhD School in Biology, University of Szeged, H6726 Szeged, Hungary; 4 Department of Surgery, University of Szeged, Semmelweis u. 8., H6725 Szeged, Hungary; furak.jozsef@med.u-szeged.hu; 5 Avicor Ltd. Alsó Kikötő sor 11/D, H6726 Szeged, Hungary; 6 Department of Physiology, Anatomy and Neuroscience, Faculty of Science and Informatics, University of Szeged, Közép fasor 52, H6726 Szeged, Hungary; 7 CS-Smartlab Devices Ltd., Ady E. u. 14., H7761 Kozármisleny, Hungary

**Keywords:** COPD, chronic airway inflammation, smoking, lung cancer

## Abstract

Chronic obstructive pulmonary disease (COPD), the frequently fatal pathology of the respiratory tract, accounts for half a billion cases globally. COPD manifests via chronic inflammatory response to irritants, frequently to tobacco smoke. The progression of COPD from early onset to advanced disease leads to the loss of the alveolar wall, pulmonary hypertension, and fibrosis of the respiratory epithelium. Here, we focus on the epidemiology, progression, and biomarkers of COPD with a particular connection to lung cancer. Dissecting the cellular and molecular players in the progression of the disease, we aim to shed light on the role of smoking, which is responsible for the disease, or at least for the more severe symptoms and worse patient outcomes. We summarize the inflammatory conditions, as well as the role of EMT and fibroblasts in establishing a cancer-prone microenvironment, i.e., the soil for ‘COPD-derived’ lung cancer. We highlight that the major health problem of COPD can be alleviated via smoking cessation, early diagnosis, and abandonment of the usage of biomass fuels on a global basis.

## 1. Background

Cancer is one of the leading deadly diseases, responsible for the death of more than half a million people in 2020, which corresponds to around 1600 deaths per day in the United States. The most common types of cancers based on gender are the following: lung cancer (23%), prostate cancer (10%), and colorectal cancer (9%) in men; lung cancer (22%), breast cancer (15%), and colorectal cancer (9%) in women [1]. Lung cancer causes 13% of all cancer deaths worldwide [2]. In 2016, the mortality rate per 100,000 person-years was 69.7 for men and 29.3 for women in Hungary. In the 5 year period between 2011 and 2016, lung cancer as a cause of death dropped by 13.9% for men, while the same number for women increased by 1.23%. This reverse change in numbers can be noticed worldwide and is due to the increased popularity of smoking amongst women [3]. Histology classifies lung cancer into two categories: small-cell lung carcinoma (15%) and non-small-cell lung carcinoma (NSCLC, 85%). Until the 1990s, squamous cell lung carcinoma was the most prevalent subtype of lung cancer, especially amongst male patients. This has now changed, whereby, as a global trend, adenocarcinoma has become the leading histologic subtype of lung cancer in both sexes, and it is responsible for more than 40% of all cases. Additionally, an increase in the overall number of lung cancer incidences in women has been observed [4]. This could be due to a preference by women to smoke cigarettes that are filtered and have a lower tar content, along with the fact that some of them are genetically predisposed to lung cancer. Environmental exposure is also a factor that should be accounted for [5]. As the second most common subtype, squamous cell lung cancer comprises 30–35% of all primary lung neoplasms. Along with small-cell lung cancer, squamous cell lung cancer is strongly associated with those having a long smoking history. To a lesser extent, large-cell lung cancer accounts for 3–5% of cases [6]. However, tobacco smoking has been described as responsible for 87% of all lung cancer-related deaths in the USA [7]. Although both types are affected differently by tobacco smoking, it has been proven that tobacco smoke is the main preliminary environmental causative factor for lung cancer. The overall 5 year survival rate is around 15% for non-small-cell lung cancer and around 6% for small-cell lung carcinoma in all stages [8]. According to information available in the eighth edition of the TNM (tumor, node, metastasis) stage system, the odds of surviving NSCLC over a period of 5 years, which vary by pathologic stage, are as follows: 73–90% in stage I, 56–65% in stage II, 12–41% in stage III, and “very poor” in stage IV [9].

Another life-threatening pathology of the lung is chronic obstructive pulmonary disease (COPD), also predominantly caused by tobacco smoking in 80–90% of patients on average [10]. The overall 5 year survival for COPD patients is between 56% and 92% depending on the severity of the disease [11]. However, according the WHO report released in 2020, COPD and lung cancer are on the list of leading causes of deaths worldwide, accounting for the third and sixth place, respectively [12]. It is also widely known that smoking generates inflammation in the airway tract via a chronic inflammatory response to irritants, frequently tobacco smoke [13]. Not only is the initiation of COPD affected by a local inflammatory reaction in the lungs, but lung cancer has also been described about leukocyte infiltrate and inflammatory reactions over the course of the disease stages [8]. It is known that oxidative stress is a causative agent for both COPD and lung cancer. Cigarettes contain approximately 10^15^ free radicals per puff including reactive nitrogen and oxygen species (RNOS). RNOS damage cells through a number of mechanisms including DNA damage (especially mitochondrial DNA) lipid peroxidation, oxidation of amino acids, and oxidation of inorganic enzyme co-factors [14]. Moreover, it is widely known that tobacco smoke consists a huge number of carcinogens (arsenic, chromium, nickel and a veritable zoo of polycyclic aromatic hydrocarbons: chrysene, methylcholanthrene, dibenzanthracene, dibenzacridene, etc.) generating mutations, which potentially make the tissue immunogenic [15]. During the inhalation of tobacco smoke, the initial response is orchestrated by the innate immune system, which includes the epithelium, mucociliary elevator, and inflammatory cells, such as macrophages and neutrophils, which protect the airways from irritants and microbes. This acute process is mainly mediated by Toll-like receptor 4 and interleukin-1 receptor signaling, which relies on the MyD88 adaptor protein. Following stimulation, this receptor complex activates the inflammasome, releasing the inflammatory interleukin-1β. The second line of defense is adaptive immunity, which is largely mediated by T lymphocytes in the respiratory tract [16]. Lung cancer and COPD may be different aspects of the same disease, with the same underlying predispositions, whether it be an underlying genetic predisposition, telomere shortening, mitochondrial dysfunction, or premature aging. In the majority of smokers, the burden of smoking may be dealt with by the body’s defense mechanisms: antioxidants such as superoxide dismutases, antiproteases, and DNA repair mechanisms. However, in the case of both diseases, these fail, frequently leading to cancer if mutations occur or COPD if damage to the cells and proteins becomes irreversible [14]. The risk of lung cancer in patients with COPD is two- to fivefold greater compared with smokers without COPD [17]. Unresolved inflammation, e.g., due to daily tobacco smoking, has been described to contribute to malignancy via the infiltration of inflammatory cells releasing inflammatory mediators such as IL-1β, interleukin-6 (IL-6), tumor necrosis factor-α (TNF-α), and RNOS [18]. Chronic inflammation is considered as one of the hallmarks of cancer, as suggested by Alberto Mantovani [19]. However, there are a myriad of hematopoietic inflammatory cells in the bronchoalveolar lymphoid tissue, and two obstacles hamper effective antitumor immunity. One obstacle is the immunosuppressive microenvironment (due to alternative polarization, established by monocytic and granulocytic myeloid-derived suppressor cells, M2 macrophages, regulatory T-cells) leading to tolerance against potentially malignant cells [20]. Another obstacle is cancer immunoediting, a concept proposed first by Screiber’s group [21,22]. Immune surveillance selects for transformed cells (elimination) but some of them can escape the immune clearance; these survivors could remain dormant up to decades (equilibrium), and further iterations of evasion mechanisms may ultimately drive immune suppression beyond the local microenvironment, accomplishing immune escape when cancer manifests [23,24,25]. There is no cure available for COPD; there are only treatments to reduce symptoms and support patient health. The clinical management of lung cancer types is complex and beyond the scope of this paper. Briefly, early diagnosis, surgery, and complex treatment through a combination of chemotherapy, radiotherapy, and targeted immunotherapy according to stage can be successful [26,27,28].

Here, we focus on COPD affecting primarily the bronchoalveolar tract and the lungs, and we shed light on the relationship between COPD and lung cancer as a consequence of chronic inflammation, initiated in most cases by tobacco smoking, which is responsible for the disease, or at least for the more severe symptoms and worse patient outcomes.

## 2. Epidemiology of COPD

The most important risk factors for chronic respiratory diseases (CRDs) have been identified and include tobacco use, exposure to indoor and outdoor pollutants, allergens, occupational exposure, unhealthy diet, obesity, physical inactivity, and other factors [29]. COPD is the third leading cause of death worldwide, after ischemic heart disease and neoplasms [30]. Chronic respiratory diseases accounted for 544 million cases throughout the globe in 2017, in which the burden of COPD shared 55.1% of cases in men and 54.8% in women [30]. The incidence of COPD was 174 million in 2015, and there were around 3.2 million deaths because of COPD worldwide [31]. The prevalence of COPD ranges between 15% and 20% of the adult population in Europe aged over 40 years [32,33]. A complex and thorough analysis based on studies conducted in almost 30 countries during a 14 year period between 1990 and 2004 allowed drawing conclusions about the chances of developing COPD, finding that it is more common in people with a smoking history [34]. There is a difference in the incidence of COPD between men and women. In the Rotterdam study, the overall incidence rate (IR) of COPD was 8.9/1000 person-years (PY); the IR was higher in men (13.3/1000 PY) than in women (6.1/1000 PY). The incidence of COPD increased from the age of 45 in both sexes. The IR was higher in current and former smokers than in never smokers: 19.7/1000 PY in current smokers, 8.3/1000 PY in former smokers, and 4.1/1000 PY in never smokers. The IR of COPD in smoking men was 15.0/1000 PY compared to 8.6/1000 PY in smoking women. The IR was 6.0/1000 PY in never smoking men and 3.7/1000 PY in never smoking women. The proportion of COPD participants without a history of smoking was 27.2% for females, while this proportion was 7.3 % in males. Taken together, there was a higher incidence of COPD in males and in smokers [35]. However, the mortality rates vary from country to country: <20 per 100,000 population in Sweden, Iceland, and Norway, but more than 80 per 100,000 population in Ukraine and Romania [36]. In developing countries, mortality is also on the rise, correlated with the increase in cigarette smoking. In China, tobacco is responsible for 12% of deaths, with projections showing that this rate could reach 33% in 2030 [37]. Not only the incidence, but also the mortality from COPD is higher in males than in females, increasing with age in those over 45 years old. The presence of COPD at various stages is associated with the following risk of death, as defined by hazard ratio: HR 2.7 for severe COPD (95% CI: 2.1 to 3.5) and HR 1.6 for moderate COPD (95% CI: 1.4 to 2.0) [38].

## 3. Risk Factors for Developing COPD

COPD is a chronic inflammatory disease of the respiratory system caused mainly by exposure to tobacco smoke, biomass fuel smoke, or polluted air with toxic gases, coal particles, or inorganic trace pollutants. While tobacco smoking is considered the main risk factor for developing COPD, only approximately 45–50% of tobacco smokers suffer from the disease [39]. Around 25–45% of COPD patients have never smoked, with more evidence showing that other risk factors, such as airway infections, starvation, chronic asthma, impaired lung growth, and poor socioeconomic status, are important for COPD development [40,41,42]. The additive effects of external risk factors such as occupational dust exposure, industrial toxic gases and vapors, and indoor burning of biomass fuels, along with inherent genetic predisposition, prenatal birth, infections of the infant, and prenatal exposure to maternal smoke, increase the risk of COPD [43]. The most frequent risk factors are the burning of biomass fuels such as animal dung, crop residues, coal, and wood for heating and cooking in low-income countries [44]. Ingebrigtsen et al. studied 22,422 Danish and 27,668 Swedish mono- and dizygotic twin pairs and found that COPD has approximately 60% heritability [45]. The study of Zhou et al. found the heritability of spirometric phenotypes according to reduced forced expiratory volume in 1 s (FEV1) and FEV1/FVC (forced vital capacity) to be around 37%, while the heritability of chest computer tomography phenotypes (emphysema) was around 25% [46]. Genome-wide association studies (GWAS) have identified genes with single-nucleotide polymorphisms (SNPs) associated with COPD such as CHRNA3/*CHRNA5* [47], *HHIP* [48], *FAM13A* [49], and *CYP2A6* [50]; these SNP susceptibility loci correlate with different phenotypes or disease severity of COPD. One of the best known genetic factors directly predisposing an individual for COPD is the deficiency of the α-1 antitrypsin (*SERPINA1* gene), causing panlobular emphysema in 1–3% of the patients [51].

## 4. Phenotypes: Many Faces of COPD

COPD is characterized by persistent respiratory symptoms and airflow limitation due to different noxae. This is caused by a mixture of small airway diseases and parenchymal destruction (emphysema). The two prototypical pathologies that result in COPD are emphysema and chronic bronchitis (CB), which typically coexist within a single patient. This determines the clinical feature. One type is “pink puffers” (predominantly emphysema) with breathlessness, hyperinflation, mild hypoxemia, and a low pCO_2_, while another type is “blue bloaters” (predominantly CB) with hypoxemia, secondary polycythemia, CO_2_ retention, pulmonary hypertension, and cor pulmonale [52]. The clinical feature is important for diagnosis. Pink puffer is type “A”, whereby coughing is rare, with scant clear, mucoid sputum; the patients are thin, with recent weight loss common. Blue bloater is type “B”, featuring a form of chronic bronchitis (CB) which is classically defined as chronic cough and sputum production for 3 months a year for two consecutive years. CB hastens lung function decline, increases the risk of exacerbations, reduces quality of life, and may increase mortality [53]. The grouping of COPD has expanded over the years according to clinical needs. In 2010, Han et al. proposed the following definition of COPD phenotypes—a single or combination of disease attributes that describe differences between individuals with COPD as they relate to clinically meaningful outcomes [54]. Today, seven main phenotypes of COPD are determined: (1) The asthma–COPD overlap phenotype occurs when two major criteria or one major and two minor criteria are met. The major criteria are a positive bronchodilator test (increase in FEV1 ≥15% predicted and ≥400 mL), eosinophilia in sputum, and personal history of asthma; the minor criteria are high total IgE, personal history of atopy, and positive bronchodilator test (increase in FEV1 ≥12% predicted and ≥200 mL) on two or more occasions. It is associated with increased frequency and severity of exacerbations, faster FEV1 decline, increased comorbid burden and mortality [55]; (2) the frequent exacerbator phenotype occurs with two or more COPD exacerbations per year, leading to up to threefold increased mortality [56]; (3) The upper lobe-predominant emphysema type occurs in patients with significant symptoms despite maximal therapy and upper lobe-predominant emphysema with CT findings, and consideration should be given to surgical lung volume reduction surgery (LVRS) [57]; (4) the ‘rapid decliner’ phenotype is accompanied by a rapid decline in lung function. The patients are relatively younger with poor nutritional status and high mortality [58]; (5) the ‘comorbid’ phenotype occurs in elder patients with moderate respiratory disease and a significant comorbid burden, predominantly cardiovascular disease, accompanied by an increased mortality risk. Subjects are characterized by a high body mass index, high rates of diabetes, congestive heart failure, and/or ischemic heart disease [59,60]; (6) the physical frailty phenotype is a multidimensional syndrome characterized by loss of physiologic and cognitive reserve, which carries prognostic significance. It is a common geriatric syndrome but does not correlate with age only. It can appear with a heavier symptom burden, frequent exacerbations, and poor functional capacity [60,61,62]; (7) the characteristics of the emotional frailty phenotype are anxiety, depression, and fear of breathlessness, which are associated with a well-documented increase in morbidity, mortality, and hospitalizations [60,63].

Morning symptoms are common in chronic obstructive pulmonary disease. Many COPD patients consider the morning as the most troublesome part of the day, during which they experience more symptoms and physical activity limitations. Physicians should consider morning symptoms as a treatment goal [64].

## 5. Diagnostic Tests of COPD

Remodeling small airways, wall thickening of the small bronchi, destruction of lung parenchyma, loss of alveolar space, and consequent decreased respiratory area are the main pathological changes in COPD. Therefore, imaging techniques (X-ray or high-resolution computer tomography) are used for diagnosis and control of pulmonary emphysema and bronchial wall thickening [65]. However, the basic diagnostic method has remained the spirometry lung function test in COPD. Patients should additionally undergo real-time, quantitative PCR (qRT-PCR) testing for SARS-CoV-2 before coughing and increasing the risk of transmission [66]. Airflow limitation is measured as a postbronchodilator FEV1 to FVC ratio <0.70, generally without reversibility to bronchodilators. At a late stage, the widespread obstruction to airflow can result in trapping of air within the lung and progressive expansion of the total lung capacity, residual volume (RV), and functional residual capacity (FRC), known as hyperinflation. These are static ventilation parameters usually determined with the co-measurement of a body plethysmograph. The Global Initiative for Chronic Obstructive Lung Disease (GOLD) has categorized the severity of airflow limitation in COPD into four groups according to the postbronchodilator FEV1 value. In mild COPD, the FEV1 is ≥80 (GOLD 1); in moderate COPD, the FEV1 is 50–80 (GOLD 2); in severe COPD, the FEV1 is 30–50 (GOLD 3); in very severe COPD, the FEV1 is <30 (GOLD 4) [67]. Medical history, physical examination, and lung function tests together give the correct diagnosis. However, there is no valid COPD diagnosis without measurable obstruction. In addition to the monitoring of dyspnea and exacerbations, an analytical investigation of volatile organic compounds in the exhaled breath can be used to diagnose COPD [68]. Another parameter for the early detection of COPD is the diffusing capacity of the lung for carbon monoxide (DLCO); subjects with normal spirometry/low DLCO developed COPD in 22% of cases versus 3% in the control (normal spirometry/normal DLCO) group [69]. Similarly, using arterial blood gas analysis, a higher ratio of paO_2_/paCO_2_ (partial pressures of oxygen and carbon dioxide in the arterial blood) is correlated with a better clinical response [70]. Impulse oscillometry (IOS) measures respiratory mechanics such as pulmonary resistance and reactance and provides relevant information about distal airway functioning. However, a major concern related to IOS is the lack of standardization and reference values [71,72]. In addition to functional tests, it is important to note the role of genetic burden, i.e., α1-antitrypsin deficiency (AATD), a frequent genetic predisposition for lung malfunctioning, thus necessitating genetic testing of AATD in suspected individuals with COPD [73]. AATD affects 0.1–0.2% of the European population, and the prevalence of COPD in individuals with AATD is around 30% [74]. Biomarkers discussed in the next section can assist in the molecular diagnosis of COPD.

## 6. Progression, Molecular Players, and Biomarkers of COPD

The progression of COPD from early onset to advanced disease leads to the loss of the alveolar wall, pulmonary hypertension, and fibrosis of the respiratory epithelium [75]. However, COPD is not uniform in terms of progression; COPD develops with typical attributes as a spectrum of disease. Some patients have relatively stable disease and others progress rapidly. Exacerbation is accompanied by breathlessness and respiratory failure, which is common with a fatal outcome. The ECLIPSE (Evaluation of COPD Longitudinally to Identify Predictive Surrogate Endpoints) cohort study confirmed a mean rate of decline in FEV1 of 33 mL/year according to observation of 2163 COPD patients. Higher rates of FEV1 decline were seen in patients with emphysema and current smokers [76]. Two or more exacerbations per year (exacerbator type) are accompanied by higher risk of future exacerbation and severe airflow limitation [77]. Lower baseline FEV1 and excessive longitudinal FEV1 decline increase the risk of mortality [78]. The mortality of COPD is increased by a higher CT emphysema index [79]. Prevalence of bronchiectasis was 30% to 60% in studies of moderate and severe COPD. There is more extensive bronchiectasis in severe COPD [80,81]. Increased mortality and coronary artery calcium score percentile show a correlation in COPD patients [82]. Various genomics approaches such as microarrays, next-generation sequencing techniques, and qRT-PCR have been used to identify possible disease-associated diagnostic markers or therapeutic targets from affected tissues and blood on the basis of specific gene expression changes for different diseases [83,84,85], including COPD [86]. Additionally, we have built an original panel to monitor lung epithelial markers using single-cell mass cytometry, which can be used for the analysis of COPD-derived lung tissue [87].

### 6.1. Circulating Biomarkers of COPD

Biomarkers of progressive COPD include a reduction in the circulating levels of Clara cell secretory protein-16 (CC-16), soluble receptor for advanced glycation end-product (sRAGE), and syndecans [88,89,90]. The serum level of syndecan-1 was significantly decreased in GOLD 3–4 and serum syndecan-4 was significantly decreased in GOLD 1–2 COPD patients [90]. Increases in the concentrations of adiponectin, c-reactive protein (CRP), fibrinogen, leukocyte count, IL-6, interleukin-8 (IL-8), TNF-α, and Chitinase-3-like protein 1 (YKL-40) have been reported with the progression of COPD [91,92]. Adiponectin is an important modulator of the inflammatory process. An increased serum level and an overexpression of adiponectin receptors have been identified on the lung parenchyma in COPD. Adiponectin has a potential role as a biomarker in emphysema progression [92]. A lower level of circulating sRAGE is associated with emphysema severity [93]. Increased IL-6 is associated with smoking burden in patients who have smoked for more than 30 pack-years. According to a meta-analysis published by Shahriary et al., the serum levels of CRP and TNF-α are increased in COPD patients compared to healthy individuals [94]. Lopez-Campos et al. found that the serum concentration of both CRP and serum amyloid A (SAA) were higher in COPD than in control subjects [95]. These are signs of systemic inflammation. High levels of CRP, TNF-α, and SAA, in conjunction with increased fibrinogen and leukocyte count, are correlated with the number of exacerbations and mortality [94]. YKL-40 is a member of the mammalian chitinase-like protein family; it is an inflammatory glycoprotein that may play an important pathogenic role in the remodeling of COPD by acting on lung fibroblasts, and its levels are correlated with the frequency of exacerbation episodes [96]. Serum YKL-40 concentration was reported to be higher in COPD than in asthma patients [97]. Other soluble mediators have been reported as biomarkers of COPD severity, such as a higher serum concentration of pulmonary and activation regulated chemokine (PARC or known as CCL18) in COPD patients compared to smoker or nonsmoker healthy controls [98]. The serum endostatin level showed a higher concentration in both stable and exacerbating COPD compared to healthy controls, which was accompanied by a positive correlation to serum CRP level [99]. The MMP-mediated degradation of type IV collagen α3 chain (C4Ma3) in the plasma of COPD patients was also present to a greater level and correlated with high mortality [100]. The acute phase protein, pentraxin-3 (PTX-3), and surfactant protein D showed a higher serum concentration in COPD patients versus healthy controls [101,102].

### 6.2. Sputum Biomarkers of COPD

The level of human neutrophil peptides (HNPs) can be used as biotracers of functional impairment in COPD. A high level of HNPs in sputum shows a negative correlation with FEV1 and FEV1/FVC ratio. A greater decline in lung function is associated with a higher amount of the following biomarkers in the sputum: matrix metalloproteinase-9 (MMP-9), neutrophil elastase (NE), and IL-8 [103]. The members of the MMP family play a role in the destruction of elastin, the remodeling of damaged alveoli, and the progression of cigarette smoke-induced COPD, especially in emphysema. NE is a serine protease secreted by neutrophils. Its concentration in the sputum is elevated in bacterial infections in patients with COPD [103]. An increased concentration of sputum IL-6, IL-8, and myeloperoxidase (MPO) can predict the frequency of exacerbation [104]. Sputum samples from COPD patients with exacerbation showed a higher content of prostaglandin D2, 12-oxo-eicosatetraenoic acid (12-Oxo-ETE), and 5-oxo-eicosatetraenoic acid (5-Oxo-ETE) [105].

### 6.3. Biomarkers of the Inflammatory Microenvironment in COPD

The involvement of neutrophils, macrophages, and NK cells in airway inflammation is an early observation in COPD [106]. Higher eosinophilic inflammation was reported in a subgroup of patients with exacerbations [107]; these patients with asthma–chronic obstructive pulmonary disease overlap syndrome (ACOS) showed a high T helper type 2 (Th2) signature and responded well to both oral and inhaled corticosteroids (ICS) [108,109]. The number of eosinophils should be above 300/µL blood for a better prognosis [110]. An inflammatory microenvironment in the airway epithelium induces inducible nitric oxide synthase (iNOS), thereby releasing NO [111]. Another biomarker of eosinophilic airway inflammation in COPD is fractional exhaled nitric oxide (FeNO) [112]. Antus et al. showed that exacerbating COPD patients with high FeNO at the time of hospitalization could serve as a predictive marker for response to therapy, showing a positive correlation with the increase in FEV1 and a negative correlation with the length of stay in hospital [113]. The exhaled breath condensate was shown to contain 2.5-fold higher concentration of Leukotriene B_4_ (LTB_4_), a potent neutrophil chemoattractant, and 2.2-fold higher concentration of Prostaglandin E_2_ (PGE2) in COPD subjects than in healthy controls [114]. Bronchial epithelial cells are the first anatomical barrier exposed to noxious particles of cigarette smoke, and they are involved in the initiation of airway remodeling through the release of several proinflammatory mediators [115]. Using a tracheobronchial epithelial cell model, Menzel et al. showed that cigarette smoke dampens the immune response against *Haemophilus influenzae* via downregulation of the NF-κB activity, which may result in higher susceptibility to infections [116]. One of the main regulators of epithelial cell function and proliferation is epidermal growth factor receptor (EGFR) signaling; smoking induces the distal to proximal rearrangement of the small airway epithelium, resulting in exaggerated EGFR signaling [117]. The upregulation and the activation of EGFR facilitate the worsening of COPD conditions, such as the production of goblet cells, mucus hypersecretion, and epithelial–mesenchymal transition (EMT) [118]. Nicotine in cigarette smoke has been reported to activate the Wnt3a signaling pathway, which results in translocation of beta catenin to the nucleus, inducing typical EMT features. Several EMT markers have been reported such as α-SMA (α-smooth muscle actin), vimentin, fibroblast-specific protein-1 (FSP-1 or S100A4), desmin, (pro)-collagen, fibronectin, connective tissue growth factor (CTGF), N-cadherin, the Snail, Slug, Twist, and beta-catenin bounded transcription factors, and the expression of MMPs [119,120]. However, a myriad of factors can induce EMT, e.g., hypoxia, reactive oxygen species (ROS), or extracellular ligands such as transforming growth factor- β (TGF-β), fibroblast growth factor (FGF), connective tissue growth factor (CTGF), and insulin-like growth factor (IGF) [121,122].

### 6.4. Extracellular Vesicles and COPD

The rapidly expanding field of EVs and a detailed discussion on the role of EVs in COPD have been reviewed elsewhere [123,124,125]. However, it is worth highlighting that EVs are involved in COPD pathogenesis via several mechanisms. Extracellular vesicles (EVs) are cell-derived microparticles surrounded by a phospholipid bilayer, and they have been reported in conjunction with several COPD attributes. EVs can be categorized as apoptotic bodies (1–4 µm), microparticles (100–200 nm), and exosomes (30–100 nm) [126]. It was shown that cigarette smoke extract induced the miR-210 content of broncho-epithelial cell-derived EVs that contributed to lung fibrosis via silencing of the ATG7 autophagy modulator [127]. Cigarette smoke extract treatment was also shown to induce other elements of epithelial EVs, such as the cleaved form of full-length CCN1 (CYR61/CTGF/NOV family1), which activates the secretion of MMP-1 and contributes to emphysema via degradation of the ECM [128]. Alveolar macrophages, as one of the major players of the inflammatory microenvironment in COPD, also produce EVs rich in cytokines, inflammatory mediators such as IL-8, MCP-1, and ICAM-1, or proteases such as MMP-14, thereby promoting emphysema by degrading collagen [129]. Activated polymorphonuclear cell (e.g., neutrophil)-derived CD63^+^/CD66^+^ exosomes coated with NE were also shown to degrade ECM components and augment the disease severity of emphysema [130]. Endothelial damage during the course of COPD development may lead to a release of endothelial EVs from activated or apoptotic cells carrying CD31, CD144, CD143, CD62E, and CD105 molecules, which exacerbate inflammation and facilitate apoptosis of neighboring endothelial cells [129,131,132]. EVs harbor cargo molecules such as lipids, miRNAs, and proteins as disease-specific agents offering diagnostic potential or representing putative therapeutic targets [133]. However, the versatile functions of various types of EVs may fuel chronic inflammation, contribute to tissue reorganization, and maintain the disturbance of tissue homeostasis, which may favor cancer development.

### 6.5. Purinergic Signaling in COPD

Several studies have reported the role of purinergic signaling (including ATP, pyrimidines, or adenosine) in airway inflammatory conditions, such as COPD [134]. The increased extracellular adenosine levels acting on P1 and elevated ATP levels acting on P2 purinergic receptors are associated with the development of COPD and exacerbation of inflammation [135,136]. All four adenosine receptors (ADORA1, ADORA2A, ADORA2B, and ADORA3) are considered to contribute to tissue injury, and they consequently serve as therapeutic targets [137]. An increased expression of ADORA2B was reported in combined pulmonary fibrosis and emphysema syndrome, and it was shown that the ADORA2B-mediated hyaluronan synthase-3 expression of macrophages led to hyaluronan accumulation, responsible for pulmonary hypertension in a mouse model [138]. The administration of an ADORA2B antagonist, GS-6201, in adenosine deaminase (ADA)-deficient animals reversed the airspace enlargement and the thickening of vascular smooth muscle wall in lung sections [139]. It was recently reported that another inhibitor, LJ-2698, an ADORA3 antagonist, restored lung function and polarized macrophages toward an anti-inflammatory state [140].

### 6.6. Galectins in COPD

Galectins, pleiotropic carbohydrate-binding lectins with versatile functions, have been intensively studied in inflammatory conditions [141]. We previously revealed the immunoregulatory role of galectin-1 in causing the apoptosis of activated T-cells [142,143], and we identified galectin-1 protein as a master regulator in the promotion of tumor vascularization [144]. Galectin-1 was shown to initiate pulmonary fibrosis via activation of FAK1 kinase in a hypoxia-dependent fashion in mice [145]. Galectin-1 showed higher expression in the small airways of non-COPD smokers, while galectin-3 was expressed in the small airways of COPD patients [146]. Galectin-3 was also reported with increased levels in the bronchoalveolar lavage fluid of patients with idiopathic pulmonary fibrosis [147]. Additionally, serum galectin-3 concentration, as a potential biomarker of acute exacerbation in COPD patients, showed a positive correlation with c-reactive protein and the serum concentration of a prohormone of brain natriuretic peptide [148]. However, in a recent follow-up study, serum galectin-3 concentration showed modest elevation in smokers with COPD and chronic bronchitis but did not differentiate stable COPD patients from those that underwent future exacerbations [149]. Another galectin, galectin-9, was shown to inhibit pathological changes in a PPE (porcine pancreatic elastase) instillation-induced COPD emphysema model via inhibition of neutrophil infiltration and MMP-9 [150].

## 7. COPD: The Soil of Lung Cancer

The link between COPD and lung cancer has been reviewed elsewhere [14,151]. Here, we dissect the role of smoking, inflammatory conditions, and carcinoma-associated fibroblasts in establishing a cancer-prone microenvironment, i.e., the soil of ‘COPD-derived’ lung cancer. COPD has been reported as an additive burden and risk factor for developing lung cancer, mainly squamous cell carcinoma, especially in smokers [152]. In a study of 136 lung cancer patients, it was shown that COPD more than fourfold increased the risk of squamous cell carcinoma [153]. Overall, the frequency of COPD ranges from 40–70% among lung cancer patients, while smoking was found in 80–90% of lung cancer cases [154]. Although 80–90% of COPD or lung cancer patients are smokers, only approximately 10% of smokers develop lung cancer and approximately 20% develop COPD.

### 7.1. Genetic and Epigenetic Factors Associated with Lung Cancer in COPD

Genetic factors, epigenetic regulation, and altered miRNA expression may explain the different susceptibility of individuals to smoking-related pathologies [155]. Genome-wide association studies found SNPs in the alpha-neuronal nicotinic acetylcholine receptor subunit (CHRNA3/5, Chr 15q25) locus responsible for increased susceptibility [155,156], while genetic variants in Hedgehog-interacting protein (HHIP, Chr 4q31) and family with sequence similarity 13 member A (FAM13A gene, Chr 4q22) loci were shown to have a protective effect against COPD and lung cancer among smokers [157,158]. In a recent study by Lee et al., two SNPs were found (rs2857210, rs2621419) in the HLA-DQB2 gene associated with susceptibility to early COPD among smokers as compared to never smokers [159]. A genetic polymorphism in the 3′ untranslated region of the VEGFR1 gene, namely, the rs7326277C (CT + CC) variant, was shown to be associated with decreased risk for COPD and lung cancer compared to the rs7326277TT genotype [160]. Epigenetic regulators, such as DNA methylation and certain miRNA circuits (miR-21, miR-200b, miR-210, and miR-let7c), are associated with lung cancer in COPD patients, leading to a decreased expression of downstream genes such as *PTEN, MARCKs, TPM-1, PDCD4, SPRY-2, ETS-1, ZEB-2, FGFRL-1, EFNA-3*, and *k-RAS* together with *P53* [161]. Other miRNAs, i.e., hsa-miR-497-5p and hsa-miR-130b-5p, were recently shown to be higher expressed in COPD patients who developed non-small-cell lung cancer [162].

### 7.2. Chronic Inflammatory Microenvironment of COPD

**Figure 1:**
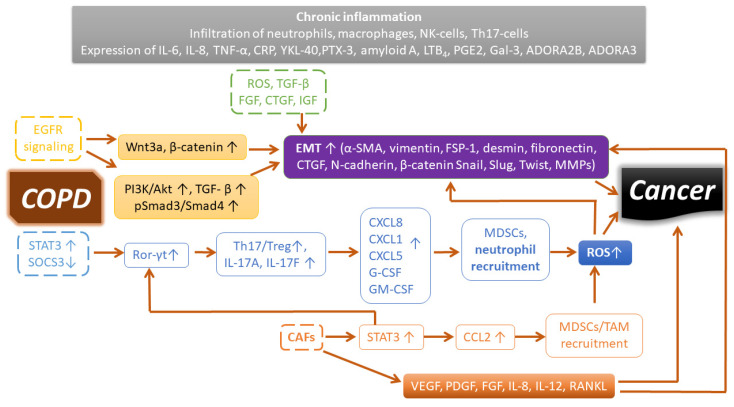
Signaling pathways and inflammatory mediators sustaining a cancer-prone microenvironment in COPD. See explanation in the text.

Deciphering genetic susceptibility is not routine and diagnosis is often late, thus limiting patient outcome. During the progress of COPD, chronic inflammation is responsible for the pathological changes in the peripheral airways and lung parenchyma [163]. There are increased numbers of polymorphonuclear neutrophil cells, macrophages, CD4 and CD8 T-lymphocytes, and B-cells in the remodeled lung in COPD, and the number of CD8 cells is also increased during disease progression. Cell counts (including neutrophils) and mediators in induced sputum are associated with lung function and risk of exacerbations [164]. Dendritic cells are localized near the surface of airways, and these detect inhaled foreign agents and facilitate innate and adaptive immunity. The release of cytokines, chemokines, lipid mediators, and growth factors by inflammatory dendritic cells, neutrophils, T-cells, epithelial and endothelial cells, and fibroblasts exacerbates airflow limitation and pulmonary fibrosis in COPD. Inflammation is mainly of the neutrophilic type in COPD; however, some patients exhibit eosinophilic inflammation with increased eosinophil cell count. This type of COPD is a good responder to corticosteroid therapy [163]. An elevated level of inflammatory lipid mediators, such as prostaglandins (PGs) and leukotrienes (LTs) released by inflammatory leukocytes in COPD, has been reported to play a role in inflammation-associated cancer development (Figure 1) [165]. Therefore, the targeting of LTs offers a potential therapeutic intervention [166]. miR-146a, as an endogenous regulator, was shown to downmodulate the production of LTB_4_ in lung cancer cells [167]. Alternatively, receptor antagonists such as the LTB_4_ receptor BLT1 antagonist, U-75302, restored SOCS1 signaling and dampened the release of inflammatory mediators in vitro [168]. An antagonist of cysteinyl leukotrienes, Zafirlukast, was also shown to improve lung function in smokers with COPD [169]. Severe COPD and frequent exacerbations are accompanied by many aspects of systemic inflammation. Elevated levels of fibrinogen, CRP, and leukocytes are associated with increased risk of comorbidities and mortality. The presence of cardiovascular disease, lung cancer, diabetes, and pneumonia can lead to a two- to fourfold deterioration in lung function [170]. The components of non-neuronal cholinergic systems, i.e., acetylcholine and cholinergic receptors, can modulate airway inflammatory cells [171]. The anti-inflammatory and bronchodilator effects of long-acting muscarinic receptor antagonists (LAMAs), such as tiotropium, were recently reviewed [172]. Emphasizing the importance of research on the tumor microenvironment focusing on the immune system, immune checkpoint blockade has shown revolutionary therapeutic achievements. Nevertheless, resistance may develop with more aggressive malignancy during relapse. A further understanding of immune tolerance is greatly needed for the development of novel therapeutics and treatments [173]. The immune system is highly plastic owing to the complexity of cellular fates in executing the versatile polarization of lymphoid and myeloid cells. Activation and polarization evolve from interactions in the network of immune cells with the surrounding soluble or anchored signals. The evolutionary advantage of this versatile polarization is the elimination of pathogenic invaders and cancerous cells. However, due to genetic and environmental factors, the polarization of macrophages (M1 or M2) and T-cells (Th1, Th2, Th17, Th22, T-regs) may be influenced and disturbed, thereby rendering the immune system ineffective against certain pathologies. We previously conducted research on the tumor microenvironment and discovered retinoic-acid-related orphan receptor RORC1/ROR-γt (retinoic-acid-related orphan receptor-γt) as a driver in cancer-related myeloid expansion [174]. Interestingly, the ROR-γt-positive cells were higher in the lung alveolar walls of COPD patients compared with normal smokers or nonsmokers [175]. ROR-γt is the key transcription factor for interleukin-17A (IL-17A) or IL-17F production, which are released by Th17 CD4^+^ lymphocytes and myeloid cells (neutrophils, eosinophils, and mast cells) [176]. IL-17 may induce the secretion of CXCL8, CXCL1, CXCL5, G-CSF, and GM-CSF from airway epithelial cells, which recruit neutrophils; thus, they may also be related to airway obstruction, emphysema, and even lung cancer development (chronic inflammation) via ROS production and tissue damage (Figure 1) [177,178,179]. Th17 development is induced by an increase in signal transducer and activator of transcription-3 (STAT3) and a decrease in its inhibitor, suppressor of cytokine signaling inhibitor-3 (SOCS3), which leads to the induction of ROR-γt, thus causing an imbalance of Treg/Th17 populations and maintaining the chronic inflammatory microenvironment that favors cancer development [180]. Additionally, IL-17 has been shown to promote NSCLC cell survival and therapeutic resistance to combined therapy of selumetinib, a mitogen-activated protein kinase kinase (MEK) inhibitor, and PD-L1 immunotherapy [181,182].

We summarized the origin and the mechanisms of tumor-promoting myeloid cells elsewhere [20], along with an inventory of drugs modulating antitumor immunity toward an effective response, which may be worth examining in exacerbating COPD [173]. Cigarette smoke may contain approximately 4800 compounds, at least 100 with a known carcinogenic effect; as such, it is not surprising that 80–90% of lung-related cancers are linked to smoking [183]. Smoking, as well as other noxious gases, dust, small particles, and oxidative agents in the air, may induce inflammation and maintain the chronic inflammatory microenvironment of the respiratory tract. Metastatic (MMPs) and angiogenetic (VEGF) factors may link EMT to the development of lung cancer [151,184]. Cigarette smoking and nicotine induce the release of active TGF-β [185], which has also been reported as one of the inducers of EMT in lung cancer [186]. EGFR-mediated PI3K/Akt activation may phosphorylate TGF-β-mediated Smad3, leading to the translocation of Smad3 along with Smad4 to the nucleus, thereby reprogramming epithelial cells toward EMT [187,188]. EMT may endow cells with a more migratory (invasive) phenotype, characterized by a loss of polarity and reorganization of the cytoskeleton. The inflammatory microenvironment induces tissue remodeling in the lung parenchyma of the airways via epithelial–mesenchymal transition, leading to lung fibrosis or lung cancer [189]. Destruction of the lung parenchyma in COPD may result in the detachment of cells into circulation, similarly to the dissemination of circulating cancer stem cells from solid tumors. An approach to identify these circulating pulmonary cells using cytokeratin and CD44v6 markers was proposed in a study by Romero-Palacios et al. in 35% of COPD patients [190].

### 7.3. Cancer-Associated Fibroblasts and COPD

The microenvironment of solid tumors consists of a variety of molecular (extracellular matrix) and cellular (tumor cells, fibroblasts, endothelial cells, pericytes, and leukocytes) elements called tumor stroma components [20]. In the tumor stroma, the alteration of cellular homeostasis is initiated not only by the transformation of tumor cells, but also by the activation of surrounding fibroblasts gaining the phenotype of cancer-associated fibroblasts (CAFs) [144,191]. It is suggested that fibroblast–epithelial interactions can facilitate EMT and tumor progression [192,193]. Cancer-associated fibroblasts produce a variety of ECM components to provide a soil for the growth of malignant cells. Cancer associated-fibroblasts nurse tumor cells by producing angiogenic factors (e.g., VEGF, PDGF, FGF), establishing an immune barrier and favoring cancer cell metastasis [194]. Cancer-associated fibroblasts have been reported to contribute to the chemoresistance of tumors in a soluble-factor-mediated or cell-adhesion-mediated manner [195]. One marker of CAFs is the overexpression of fibroblast activation protein (FAP) in >90% of human carcinomas [196]. It was shown that FAP activation and its associated STAT3–CCL2 signaling in cancer-associated fibroblasts recruited immunosuppressive myeloid-derived suppressor cells and tumor-associated macrophages [20,173,197]. A high expression of FAP has been reported as a prognostic factor with worse patient prognosis and disease progression in lung adenocarcinoma [198]. Normalization of the tumor microenvironment through the therapeutic targeting of FAP-expressing cells has been successfully addressed in several studies using animal models [199,200]. Indeed, fibroblasts may represent therapeutic targets in COPD since it has been shown that cigarette smoke alters the proteomic profile of lung fibroblasts through the differential expression of genes in mitochondrial activity and cellular aging [201]. Another group recently showed the senescent phenotype of severe, early-onset COPD lung fibroblasts with higher expression levels of chronic inflammatory proteins such as IL-8, interleukin-12 (IL-12), and receptor activator of nuclear factor kappa-Β ligand (RANKL) [202,203].

### 7.4. Lung Microbiota and COPD

The lung was previously considered sterile. However, studies using next-generation sequencing of bacterial 16S rRNA revealed the existence and complexity of the microbiota in both the lower and the upper respiratory tract, even in physiological conditions [204]. Noxious gases, such as tobacco smoke, dampen the innate immune defenses, which may lead to a change in lung microbial composition, thus potentially resulting in greater inflammation during the course of COPD [205]. Proteobacteria such as *Moraxella* and *Haemophilus* are reported with increased abundance, while Firmicutes such as *Streptoccoccus* species are reported with decreased abundance in COPD exacerbations [206]. An analysis of 101 sputum samples revealed that neutrophilic inflammation was associated with *Haemophilus*, while the host interferon response was correlated with *Moraxella* abundance [207]. An alteration of gut microbiota and metabolites was also reported in COPD, whereby 146 gut bacterial species were associated with COPD with an elevated ratio of *Streptococcus* spp. and *Lachnospiracea*; furthermore, from the investigated top 50 fecal metabolites, 46% of lipid- and 20% of amino-acid-related metabolites showed a COPD signature [208]. Alteration of the commensal microbiota may trigger chronic pathogen stimuli, invade the mucosal surface, and maintain the inflammatory microenvironment, i.e., the soil for lung cancer [209]. The abovementioned *Streptococcus* and *Moraxella*, as well as *Pseudomonas, Staphylococus,* and *Veillonella*, are widely reported as lung-cancer-associated bacteria [209]. An imbalance of the composition of the lung microbiota may (i) modulate the oncogene activation of the host via bacterial metabolites, and (ii) fuel neutrophilic inflammation and suppress antigen-specific tumor clearance [210].
